# AI-driven quality classification of concrete after high-temperature exposure: a hybrid framework for structural integrity assessment

**DOI:** 10.1038/s41598-026-52318-9

**Published:** 2026-05-19

**Authors:** Wonchang Kim, Taegyu Lee

**Affiliations:** Department of prevention fire and disaster, Semyung University, Jecheon-si, 27122 South Korea

**Keywords:** Concrete, High-temperature, Artificial intelligence, Machine learning, Non-destructive test, Engineering, Mathematics and computing

## Abstract

Accurately assessing the residual performance of fire-damaged concrete is crucial for structural safety but challenging due to non-linear degradation. Traditional methods often suffer from data scarcity and lack objective quality criteria, creating a prediction-decision gap. This study introduces a novel AI-driven quality classification framework, shifting from numerical prediction to actionable decision-making. We established a comprehensive database (334 data points) integrating compressive strength and ultrasonic pulse velocity (UPV). To address data scarcity, the SMOTE algorithm expanded the dataset to 3,006 points. Our key innovation is a hybrid AI approach: K-means clustering identified inherent data patterns, followed by a Decision Tree (DT) to establish objective, rule-based criteria for four quality grades (Safety, Caution, Warning, Danger). Six machine learning models were rigorously evaluated using an independent test set (80/20 split). While Gradient Boosting achieved the highest accuracy (97.6%), the DT model was optimal, balancing high accuracy (96.1%) with superior interpretability (white-box model) and computational efficiency. UPV and temperature were confirmed as the dominant factors. This framework provides a reliable and practical tool for the immediate assessment of fire-damaged concrete structures.

## Introduction

Concrete undergoes severe physicochemical degradation when exposed to high temperatures^1–3^. High temperatures decompose calcium-silicate-hydrate (C-S-H) gel and calcium hydroxide, weakening the cement paste and increasing porosity^4–6^. Accurately evaluating the residual performance of fire-damaged structures is essential for safety diagnostics and repair decisions. However, the relationship between material properties, heating history, and residual performance is highly complex and non-linear.

**Fig. 1:**
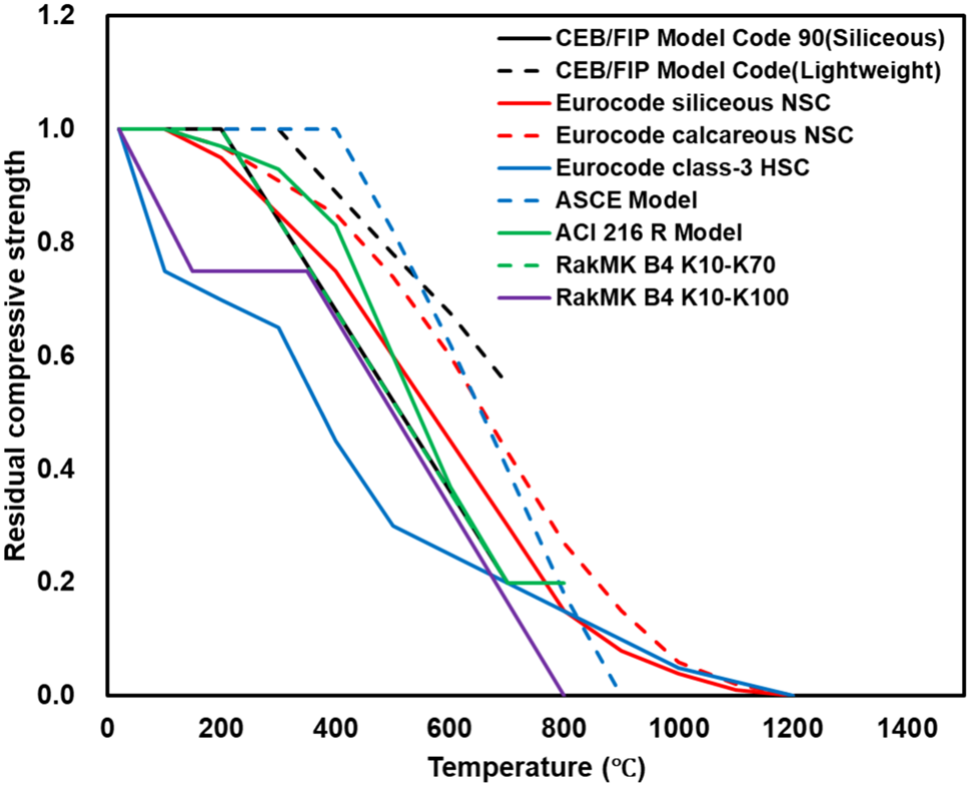
Code provisions for the residual compressive strength of concrete exposed to high temperatures.

**Fig. 2:**
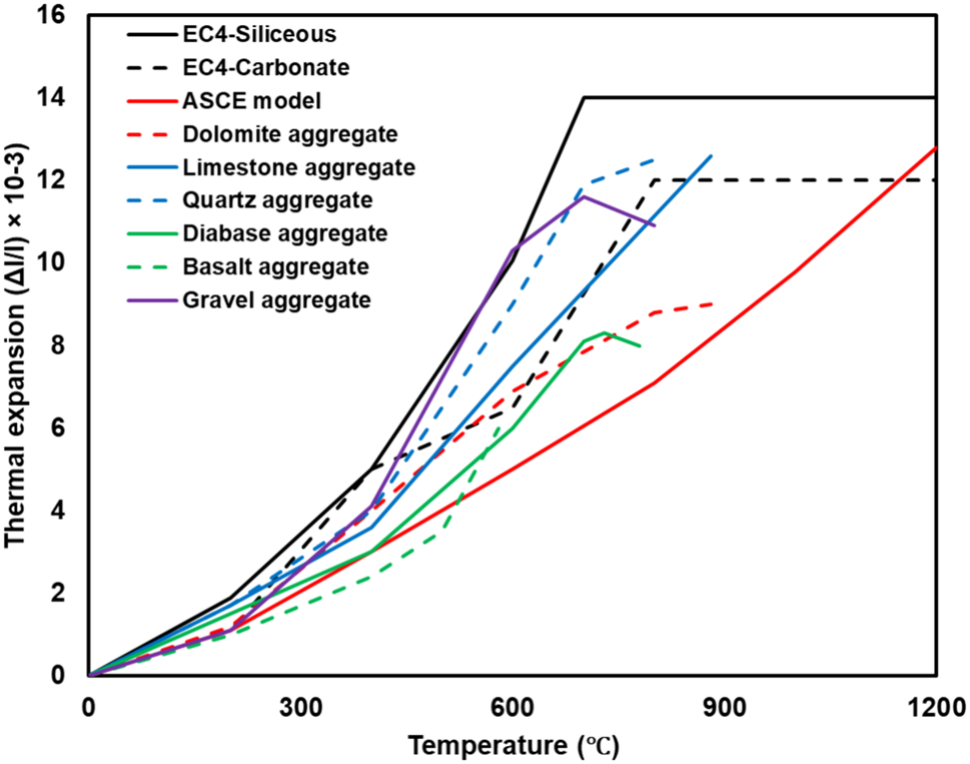
Code provisions for the thermal expansion of aggregate.

Traditionally, engineers rely on standardized codes (e.g., ACI, ASCE, CEB/FIP), which provide models relating temperature exposure to strength loss (Fig. 1) under standardized scenarios. However, the practical application of these codes is limited. As Fig. 1 illustrates, models exhibit discrepancies. Concrete performance at high temperatures is influenced by numerous variables often simplified in these models. A critical factor is the role of aggregates, as their thermal expansion coefficients vary significantly depending on mineralogy (Fig. 2). Thermal incompatibility between the aggregate and cement paste generates internal stress, accelerating microcrack formation^7^. Since organizations propose varying models, and actual behavior depends on the specific aggregate, standardized codes struggle to provide accurate assessments for diverse concrete mixes.

The reliance on these simplified, deterministic models fails to capture the highly non-linear interactions during heating. To overcome these limitations, a data-driven approach utilizing advanced computational methods is necessary. Artificial intelligence (AI) and machine learning (ML) are emerging as powerful tools for diagnosing fire damaged concrete by learning complex patterns from data^8–10^. Developing a generalized model requires a comprehensive dataset reflecting various conditions. This study compiled data from 33 independent research papers to learn these complex relationships across diverse materials. However, high-temperature experiments are resource-intensive, leading to inherent data scarcity. Limited datasets increase the risk of overfitting and decrease generalization performance^11,12^. Therefore, systematic data augmentation techniques are essential to ensure the AI model is robust and resistant to noise or outliers inherent in experimental data.

Furthermore, there is a distinct absence of objective quality classification criteria specifically for concrete after high temperature exposure. While ultrasonic pulse velocity (UPV) is a widely used non-destructive testing (NDT) method, existing UPV-based criteria (Table 1) are inappropriate for fire damaged concrete^13,14^. These guidelines were developed for sound concrete at room temperature. High temperature exposure fundamentally alters the microstructure (porosity, microcracking, chemical decomposition), drastically changing the correlation between UPV and strength^15^. Applying room temperature standards leads to unreliable and potentially unsafe assessments^16^.

**Table 1:** Conventional guidelines for concrete quality classification based on UPV ranges.

UPV range(m/s)	Concrete quality
More than 4500	Excellent
3600 – 4500	Good
3000 – 3600	Questionable
2100 – 3000	Poor
1800 – 2100	Very poor
Less than 1800	Significant anomalies are to be anticipated inside the structure

Moreover, existing research often focuses on regression-based prediction of numerical strength values. This creates a prediction decision gap, where the predicted value does not directly inform engineering actions. Practical application requires a model that translates measurable data into actionable quality classifications. While detailed data on thermal conductivity or expansion coefficients are valuable for microstructural analysis, they are difficult to obtain in field assessments and were not consistently reported in the compiled dataset. The exclusion of these parameters does not undermine the proposed framework’s validity. The objective is to develop a practical, rapid assessment framework utilizing readily obtainable field data (UPV, temperature, residual strength). The collected data inherently captures the macroscopic outcomes resulting from complex underlying thermal mechanisms. The framework can reliably classify concrete quality without explicitly modeling the underlying physics, remaining within the scope of practical engineering application^7,14,17^. To address these challenges, the inaccuracy of generalized codes, data scarcity, the lack of specialized quality criteria, and the prediction-decision gap, this study proposes a novel AI based quality classification framework^18^. We shift the research paradigm from numerical prediction to actionable classification (e.g., Safety, Caution, Danger)^19^. Data scarcity is addressed by systematically collecting literature data and applying the synthetic minority over-sampling technique (SMOTE), expanding the dataset from 334 to 3,006 points and enhancing model robustness. A unique hybrid AI approach combines unsupervised learning (K-Means clustering) to identify inherent quality clusters and supervised learning (Decision Tree) to establish objective, interpretable, rule-based classification criteria ^19^. Finally, six ML classification models are systematically compared to identify the optimal balance of accuracy, efficiency, and interpretability for practical application^12,21,22^.

## Methods

### Research framework

**Fig. 3:**
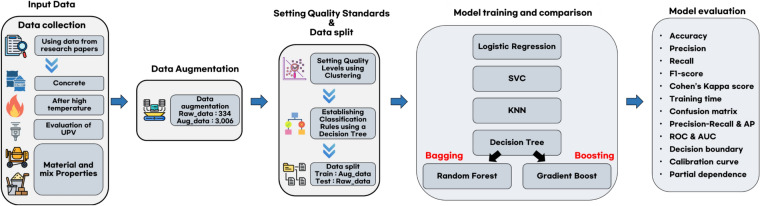
Comprehensive research framework for the AI-based quality classification of conc rete after high temperature exposure.

The comprehensive research framework for the AI-based quality classification is summarized in Fig. 3. The methodology involved five main steps:

- Step 1: Collection of comprehensive data (mix design, temperature, strength, UPV) via systematic literature review and preprocessing.
- Step 2: Application of a hybrid AI approach. Unsupervised clustering identified optimal quality grades, followed by supervised learning (DT) to derive interpretable, rule-based criteria.
- Step 3: Utilization of the SMOTE algorithm for data augmentation.
- Step 4: Development and optimization of six ML classification models (Logistic Regression (LR), Support Vector Classifier (SVC), K-Nearest Neighbors (KNN), Decision Tree (DT), Random Forest (RF), Gradient Boosting (GB)).
- Step 5: Rigorous evaluation of the models using an independent test dataset and cross validation.

### Data collection

To maximize generalization, a comprehensive database was built through a systematic literature review. The database strictly included studies where both compressive strength and UPV were simultaneously measured, essential for establishing reliable quality criteria. A total of 334 unique experimental data points were secured from 33 independent research papers (Table 2). The dataset encompasses diverse materials (normal, lightweight, heavyweight concrete) and conditions (various aggregates, W/C ratios, temperatures up to 1000°C). The scope of data collection was strictly limited to concrete using cement as the sole binder to analyze the effect of aggregate density. Consequently, special concrete types such as Ultra-High Performance Concrete (UHPC), Fiber-Reinforced Concrete (steel, polypropylene, glass), Self-Compacting Concrete (SCC), and Reactive Powder Concrete (RPC) were excluded. Therefore, the reliability of the proposed model has not been verified for these specific materials.

**Table 2:** Summary of data.

Researcher	Types of coarse aggregate	Types of fine aggregate	Specific gravity of cement	Fineness of cement	Unit weight of cement	Unit weight of water	Unit weight of coarse aggregate	Unit weight of fine aggregate	Sand/aggregate ratio
^54^	Basaltic, Expanded clay		3.1	3670	400	200	562	1066	0.65
^42^	Granite		3.15	3990	455	215	870	840	0.49
^53^	Granite				390	129	1194	710	0.37
^52^	Gravel, Shale ceramsite				468	178, 140	856, 325	856, 732	0.50, 0.69
^51^	Limestone		3.16	3650	500	100	1180.1	792.76	0.4
^50^	Limestone, Barite, Siderite	Limestone, barite	3.11	3355	450	180	791, 1225, 1086	955, 1448, 1434	0.55, 0.54, 0.57
^49^	Limestone, Electric arc furnace slag	Limestone			370	185	994, 1247	813	0.45, 0.39
^48^	Natural	Natural	3.05	3220	436	188	1309	655	0.33
^47^	Natural, Recycled	Natural	3.15		429	208, 241	973, 832	725, 747	0.43, 0.47
^46^	Palm oil clinker, Oil palm shell	Natural	3.14	3510	470	166	565, 373	846	0.60, 0.69
^45^	Pumice	Pumice	3.11	3912	400	207	448	914	0.67
^44^	Natural, Recycled	Natural	3.12	3225	320	160	1280	640	0.33
^43^	Granite	Sea	3.15	3200	336, 475, 589	185, 165, 160	956, 905, 741	797, 755, 618	0.45
^42^	Granite	Natural	3.15	3990	380	186	1320	545	0.29
^41^	Limestone	River	3.08	3996	550	182	778	869	0.53
^40^	Limestone				360	169, 122	932	931	0.5
^39^	Gravel	Natural			448	197	1182	656.8	0.36
^38^	Natural	Natural	3.14	3200	500	190	673	780	0.54
^37^	River gravel, Crushed stone"								
^36^	Crushed stone	Natural			392, 354	227, 241	914	723	0.44
^35^	Crushed stone	Natural	3.1		340	163	1356	682	0.33
^34^	Crushed stone	Natural	3.15	2900	500	170	833	916.8	0.52
^33^	Limestone, Expanded clay I Expanded clay II, Expanded shale	Natural	3.14		426	192	369, 585, 547, 1002	777	0.68, 0.57, 0.59, 0.44
^32^	Crushed stone	Crushed stone	3.12	4126	300	180	943	942	0.5
^31^	River gravel	Natural	3.1		400	200	560	1043	0.65
^30^	Limestone								
^29^	River gravel	Natural			492, 526	197, 142	984, 1052	738, 789	0.43
^28^	Siliceous	Natural			367	184	1083	716	0.4
^27^	Limestone	Crushed stone	3.23		600	192	792	792	0.5
^26^	Natural	Natural	3.12	3100	417	188	1127	641	0.36
^25^	Basalt	Natural	3.15		407	163	108	851	0.44
^24^	Crushed stone	Natural	3.15		335	156	879	936	0.52
^23^	Granite	Crushed stone	3.14	3200	400, 500, 600	165	938, 762, 896 530, 533, 507	799, 711, 676 799, 733, 676	0.46, 0.43

Density of fine aggregate	Absorption of fine aggregate	Density of coarse aggregate	Absorption of coarse aggregate	Max size of coarse aggregate	Water/cement ratio	Range of temperature	Heating rate	Size and shape of specimens	Evaluation item
2480	4.1	2560, 430	1.70, 20.10	8	0.5	200 - 1000	8	71 Cube	Fc, ML, UPV
2600	0.7	2700	0.5	10	0.47	200 - 800	20	100 Cube	ML, UPV, Fc
2160	0.35	2710	0.48	20	0.33	200 - 800	5	100 x 200 Cylinder	ML, Fc, UPV
		769	12	15	0.3, 0.38	100 - 1000	Oct-15	100 Cube	ML, Fc, UPV
		2700		17	0.2	250 - 750	5	100 Cube	UPV, Fc
2510	2.00, 0.94	2710, 4050, 3610	0.18, 1.11, 0.62	11.2	0.4	300 - 600	11	100 Cube	UPV, Fc
2640, 4110		2650, 3330	0.72, 2.67	16	0.5	400 - 800	Laboratory	150 Cube	Fc, UPV
	0.67	2660	0.73	19	0.43	300 - 800	4.5	150 x 300 Cylinder	Fc, UPV
2610	2.44	2830, 2410	1.29, 6.50	12.5	0.45	100 - 500	5	100 x 200 Cylinder	ML, Fc, UPV, E
2670	1.17	1800, 1220	19.1	3.56, 23.52	0.35	150 - 500	16.67, 8.11	100 Cube	ML, Fc, UPV
2890				16	0.5	200 - 600	5	100 Cube	ML, Fc, UPV
	0.8	2610, 2340	0.88, 5.00	19	0.5	200 - 600	3	100 x 200 Cylinder	UPV, ML, Fc
2650	1	2620	0.8	20	0.55, 0.33, 0.19	100 - 700	1	100 x 200 Cylinder	Fc, E, UPV
2650	0.7	2700	0.5	38	0.5	200 - 600	10	100 x 200 Cylinder 150 x 300 Cylinder	ML, UPV, Fc
2600	1.44	2730	0.22	16	0.33	200 - 800	1	100 Cube	ML, UPV, Fc
2590				16	0.47, 0.34	200 – 800	3	100 Cube 100 x 100 x 400 Prism	UPV, Fc
	0.98	2590	0.69	20	0.44	200 - 1000	5	150 x 300 Cylinder	ML, Fc, E, UPV
2700	1.73	2680	0.47	19	0.38	110 - 600	5	150 Cube	Fc, ML, UPV
2630				25	0.45	200 - 800	Laboratory		ML, Fc, UPV
		2600		12	0.58, 0.68	400 - 600	2.5	100 x 200 Cylinder	UPV, Fc
2620, 2720	1	2880	0.5	20	0.48	200 - 800	10	100 Cube	ML, Fc, UPV
2580	2.3	2600	1.8	12.5	0.34	300 - 700	1	100 Cube	Fc, UPV, ML
2570	2.6	2710, 824, 1346, 1320	0.51, 28.0, 24.60, 8.50	10, 8	0.45	150 - 600	1	110 x 220 Cylinder	ML, UPV, Fc, E
2600	3	2740	1	16	0.6	200 - 800	Laboratory	100 Cube	Fc, UPV
2500	3.1	2500	3.1	8	0.5	400 - 800	2.5	100 Cube	Fc, UPV
					0.45	100 - 600	5	100 x 200 Cylinder 150 x 300 Cylinder	Fc, Ft, Fs,E
				10	0.4, 0.27	200 - 800	4	100 Cube	Fc, UPV
				19	0.5	200 - 600	27.4	100 x 200 Cylinder	Fc, UPV
2680	2	2680	0.5	15	0.32	200 - 1200	1	100, 150, 200 Cube	Fc, UPV, Ft
2630	1.76	2680	0.48	20	0.45	50 - 300	0.5	100 x 200 Cylinder	Fc, UPV
2690		2740		20	0.4	200 - 1000	05-Jun	150 Cube	Fc, Fs, Ft, UPV
2600		2640		19	0.6	200 - 8000	Laboratory	100 x 200 Cylinder	Fc, UPV, Ft
2540	1.6	2680, 1470	0.68, 8.68	20	0.41, 0.33, 0.28	100 - 700	1	100 x 200 Cylinder	Fc, UPV, E

### Data preparation and preprocessing

Systematic preprocessing refined the raw data for model training. Outlier detection utilized the interquartile range (IQR) method : 16 in unit_water, 17 in unit_fine, 9 in heat_rate, and 2 in compressive strength. These were replaced with the mean value of their respective groups (categorized by aggregate type and W/C ratio). This group based mean imputation ensures that the physical characteristics of specific mix designs are preserved without the distortion associated with global replacement. Data scaling ensured equal contribution of all variables, particularly for distance-based algorithms. Min-max scaling normalized all input variables to a range between 0 and 1 (Eq. 1). This normalization enhances learning stability and predictive performance^55^.

1 $${X}_{norm}=\frac{X-{X}_{min}}{{X}_{max}-{X}_{min}}$$

Here, ${X}_{norm}$ is the normalized value, $X$ is the original value, and ${X}_{min}$ and ${X}_{max}$ are the minimum and maximum values of the respective variable.

### Data augmentation(Smote)

ML model performance depends heavily on data quantity and quality. Data augmentation is essential to overcome data scarcity, make the model robust to variations, and improve generalization^16^. This study employed the SMOTE algorithm. SMOTE generates synthetic data through interpolation, rather than simple duplication, enhancing the model’s ability to learn the decision boundary and increasing resilience to outliers. The SMOTE algorithm selects a minority class sample (${x}_{i}$) and a random neighbor (${x}_{j}$). A new synthetic data point (${x}_{new}$) is generated by linear interpolation between them (Eq. 2).

2 $${x}_{new}={x}_{i}+\lambda \times ({x}_{j}-{x}_{i})$$

Here, λ is a random number between 0 and 1. SMOTE application is crucial as the continuous nature of concrete properties makes interpolated data physically plausible. It is particularly important for balancing scarce data in extreme high temperature ranges^56^. The dataset was expanded from 334 to 3,006 points. Crucially, to prevent the generation of physically unrealistic data, this study adopted a hybrid augmentation approach. While the input features were generated using SMOTE, the target variable (residual strength) was determined using an XGBoost regressor trained on the original dataset, ensuring non-linear thermo-mechanical relationships were preserved. Subsequently, a physical plausibility filter was applied to remove any samples violating established degradation trends. To prevent overfitting caused by manual tuning, the SMOTE algorithm and the XGBoost regressor (for target prediction) were applied using default parameters.

### Hybrid AI approach for quality standards

To establish objective quality standards, a hybrid approach was used. First, K-Means clustering (unsupervised learning) was applied to the key variables (residual strength ratio, temperature, UPV) to identify inherent groupings in the data. The optimal number of clusters (k) was determined by minimizing the within cluster sum of squares (WCSS, Eq. 3), also known as the elbow method, and maximizing the silhouette score.

3 $$WCSS=\sum_{i=1}^{k}\sum_{x\in {C}_{i}}{\Vert x-{\mu}_{i}\Vert }^{2}$$

Here, $k$ is the number of clusters, ${C}_{i}$ is the $i$-th cluster, $x$ is a data point in cluster ${C}_{i}$, and ${\mu}_{i}$ is the center point of cluster ${C}_{i}$.Once the data points were labeled according to the optimal clusters (k=4), a DT(supervised learning) model was trained using these labels. DT was selected for its ability to generate interpretable, rule-based criteria. The max_depth hyperparameter was optimized to balance model complexity and prevent overfitting.

### Model development and evaluation

Six ML models (LR, SVC, KNN, DT, RF, and GB) were developed. Crucially, to strictly validate generalization capability on unseen materials, the test set was composed exclusively of original raw data isolated prior to augmentation. SMOTE was applied solely to the training set, ensuring that the evaluation reflects performance on real-world experimental conditions rather than synthetic data. Evaluation utilized comprehensive metrics (Table 3) to verify accuracy, reliability, generalization performance, and efficiency. Models were evaluated using 10-fold stratified cross validation to ensure balanced class distribution in every fold. Basic accuracy and efficiency were evaluated using accuracy, precision, recall, f1-Score, cohen’s kappa, confusion matrix, and training time. Generalization and reliability were verified using ROC curve/AUC score and precision-recall curve/AP score. Interpretability was analyzed through decision boundary visualization, calibration curves, learning curves, and partial dependence analysis.

**Table 3:** Summary of evaluation metrics used in this study.

Evaluation metrics	Purpose of evaluation	Formula/decision
Accuracy	Measures overall prediction correctness	$\frac{TP+TN}{TP+TN+FP+FN}$
Precision	Measures the accuracy of positive predictions	$\frac{TP}{TP+FP}$
Recall	Measures the model’s ability to identify all actual positives.	$\frac{TP}{TP+FN}$
f1-score	The harmonic mean of Precision and Recall ; useful for imbalanced classes	$2\times \frac{Precision\times Recall}{Precision+Recall}$
Cohen’s kappa	Measures inter-rater agreement, corrected for chance	$K=\frac{{p}_{o}-{p}_{e}}{1-{p}_{e}}$
Training time	Measures the computational efficiency of the model.	
confusion matrix	Provides a detailed breakdown of correct and incorrect classifications per class	
ROC curve & AUC score	Evaluates the model’s ability to distinguish between classes across all thresholds	AUC (Area under the ROC curve)
Precision-recall curve & AP score	Evaluates model performance on imbalanced datasets	AP (Average precision)
Feature importance	Identifies the contribution of each variable to the model’s predictions	
Decision boundary	Visualizes how the model separates classes in the feature space	
Calibration curve	Assesses the reliability of the model’s predicted probabilities	
Learning curve	Diagnoses model performance issues like overfitting or underfitting	

## Results and discussion

### Exploratory data analysis and variable

**Fig. 4:**
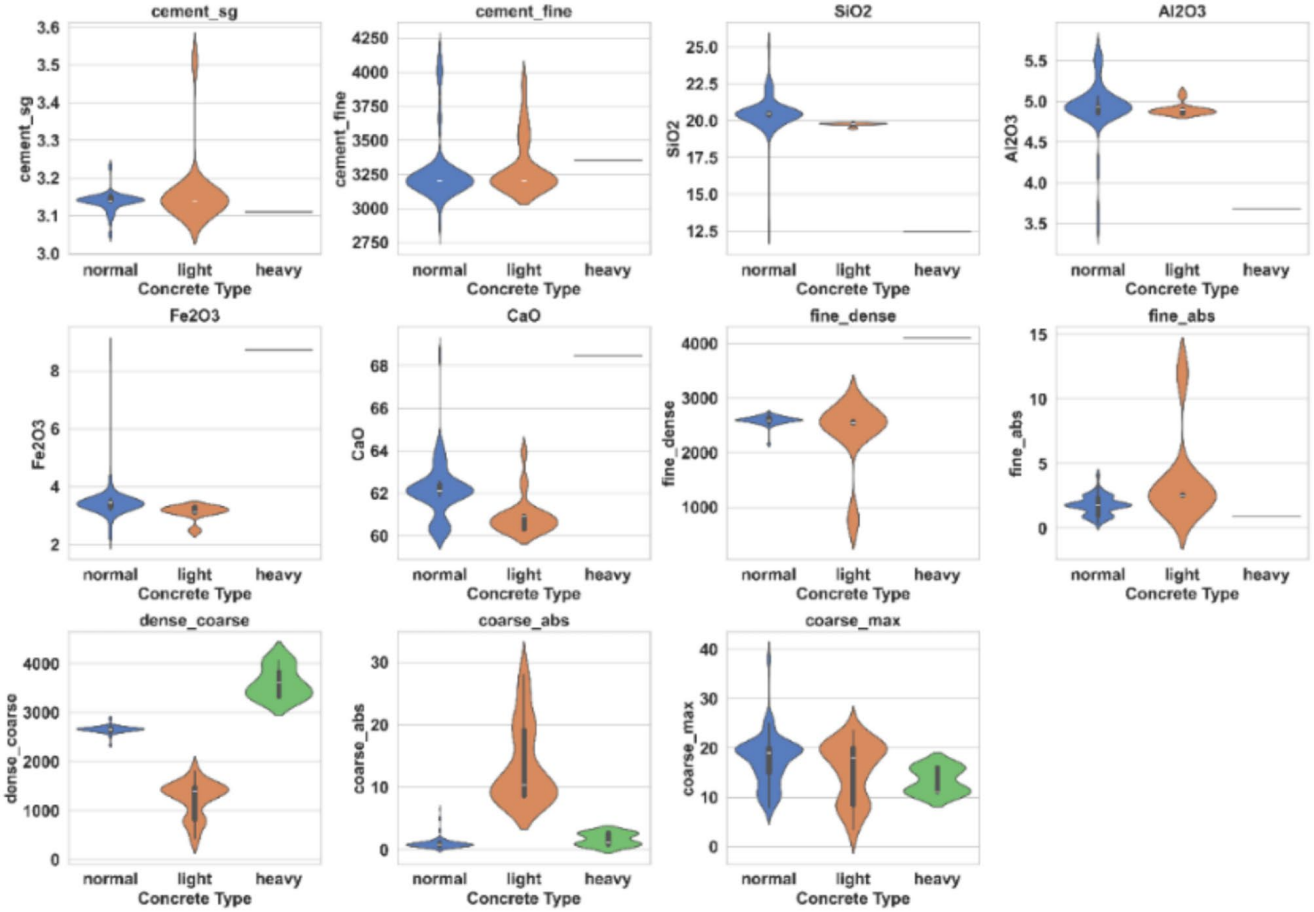
Analysis results of material properties by concrete type.

**Fig. 5:**
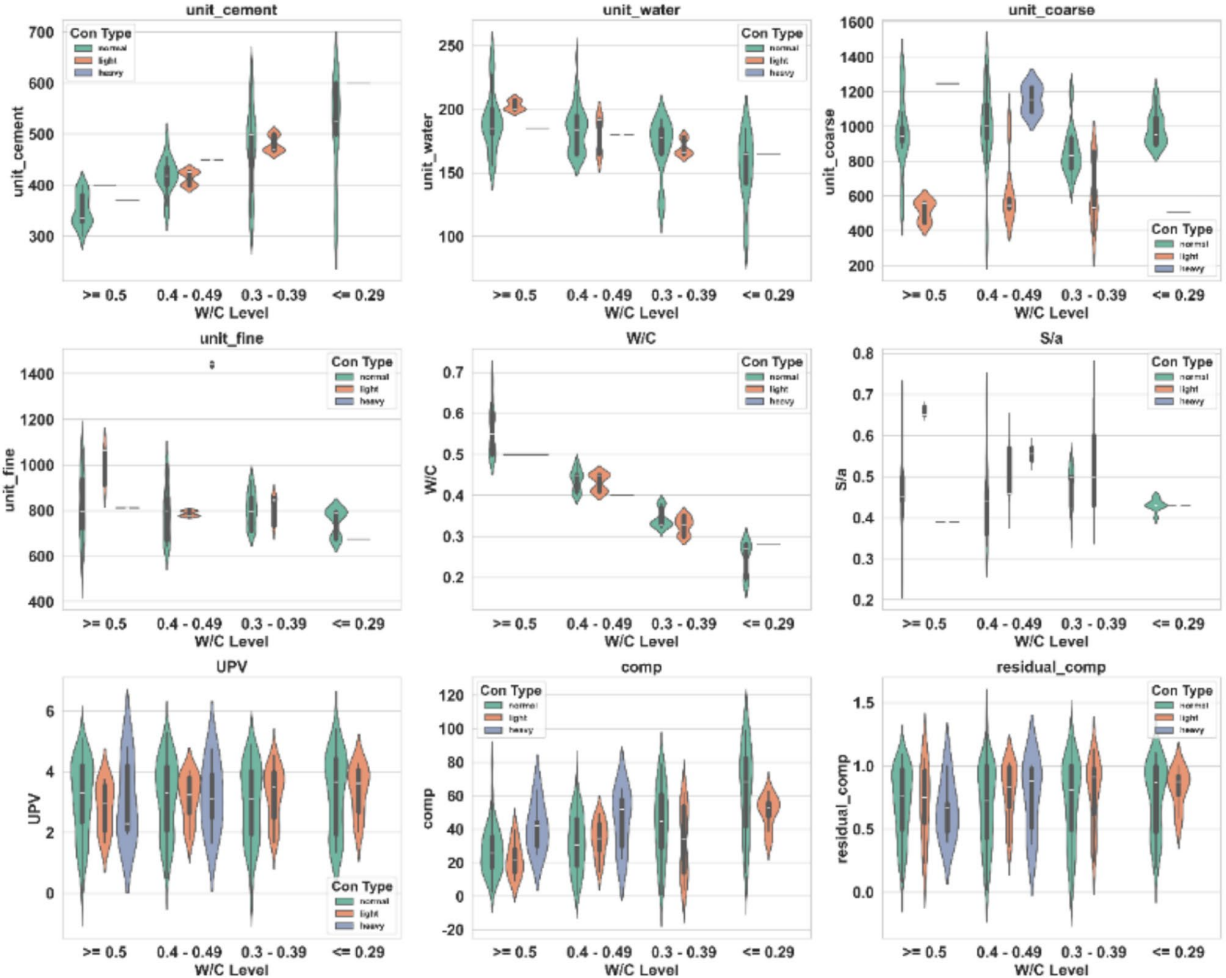
Analysis results of mix and mechanical properties of concrete by W/C range.

Exploratory data analysis(EDA) was conducted to understand data characteristics and validate the modeling strategy. The database analysis (Fig. 4/Table 4 and Fig. 5/Table 5) examined material properties based on concrete type and water/cement (W/C) ratio.

**Table 4:** Results of descriptive statistics and ANOVA for each concrete type.

Variables		Normal concrete		Lightweight concrete		Heavyweight concrete		F-statistic	p-value
		Mean	Std	Mean	Std	Mean	Std		
Cement	Specific gravity	3.14	0.03	3.16	0.09	3.11	-	9.27	< 0.05
	Fineness	3277.9	241.8	3306.9	204.2	3355.0	-	0.98	0.35
	SiO_2_	20.50	1.28	19.75	0.10	12.48	-	287.34	< 0.05
	Al_2_O_3_	4.94	0.33	4.90	0.07	3.69	-	102.85	< 0.05
	Fe_2_O_3_	3.46	0.65	3.12	0.25	8.72	-	480.16	< 0.05
	CaO	62.10	1.24	60.93	0.88	68.50	-	214.16	< 0.05
Fine_agg	Density	2594.0	81.3	2346.2	635.2	4110.0	-	146.22	< 0.05
	Absorption	1.78	0.73	3.77	3.32	0.94	-	42.29	< 0.05
Coarse_agg	Density	2655.4	73.4	1237.3	365.2	3602.7	311.8	1788.60	< 0.05
	Absorption	1.01	0.85	13.61	6.31	1.69	0.96	474.67	< 0.05
	Max size	17.54	5.13	15.40	5.90	13.38	2.51	7.57	< 0.05

**Table 5:** Results of descriptive statistics and ANOVA for each concrete type.

Variables		W/C : ≥ 0.5		W/C : 0.4 – 0.49		W/C : 0.3 – 0.39		W/C : ≤ 0.29		F-statistic	p-value
		Mean	Std	Mean	Std	Mean	Std	Mean	Std		
Unit weight	Cement	358.85	32.59	419.58	31.24	472.46	66.50	529.30	96.89	110.78	< 0.05
	Water	191.70	21.03	182.91	16.57	170.05	17.86	157.83	22.62	39.44	< 0.05
	Coarse aggregate	919.95	254.58	923.69	269.30	773.38	184.74	863.57	229.12	8.26	< 0.05
	Fine aggregate	841.16	148.96	824.11	178.26	806.93	68.56	731.78	58.96	7.14	< 0.05
	Ratio of S/a	0.48	0.11	0.46	0.09	0.49	0.07	0.43	0.01	6.33	< 0.05
	Ratio of W/C	0.54	0.06	0.43	0.03	0.34	0.02	0.26	0.04	798.96	< 0.05
Mechanical properters	Compressive strength	27.23	14.10	33.39	15.58	39.97	21.54	60.62	22.70	35.71	< 0.05
	Residual compressive strength	0.70	0.27	0.72	0.29	0.74	0.30	0.77	0.25	0.76	0.52
	UPV	3.06	1.20	3.17	1.12	3.06	1.13	3.33	1.16	0.71	0.54

**Fig. 6:**
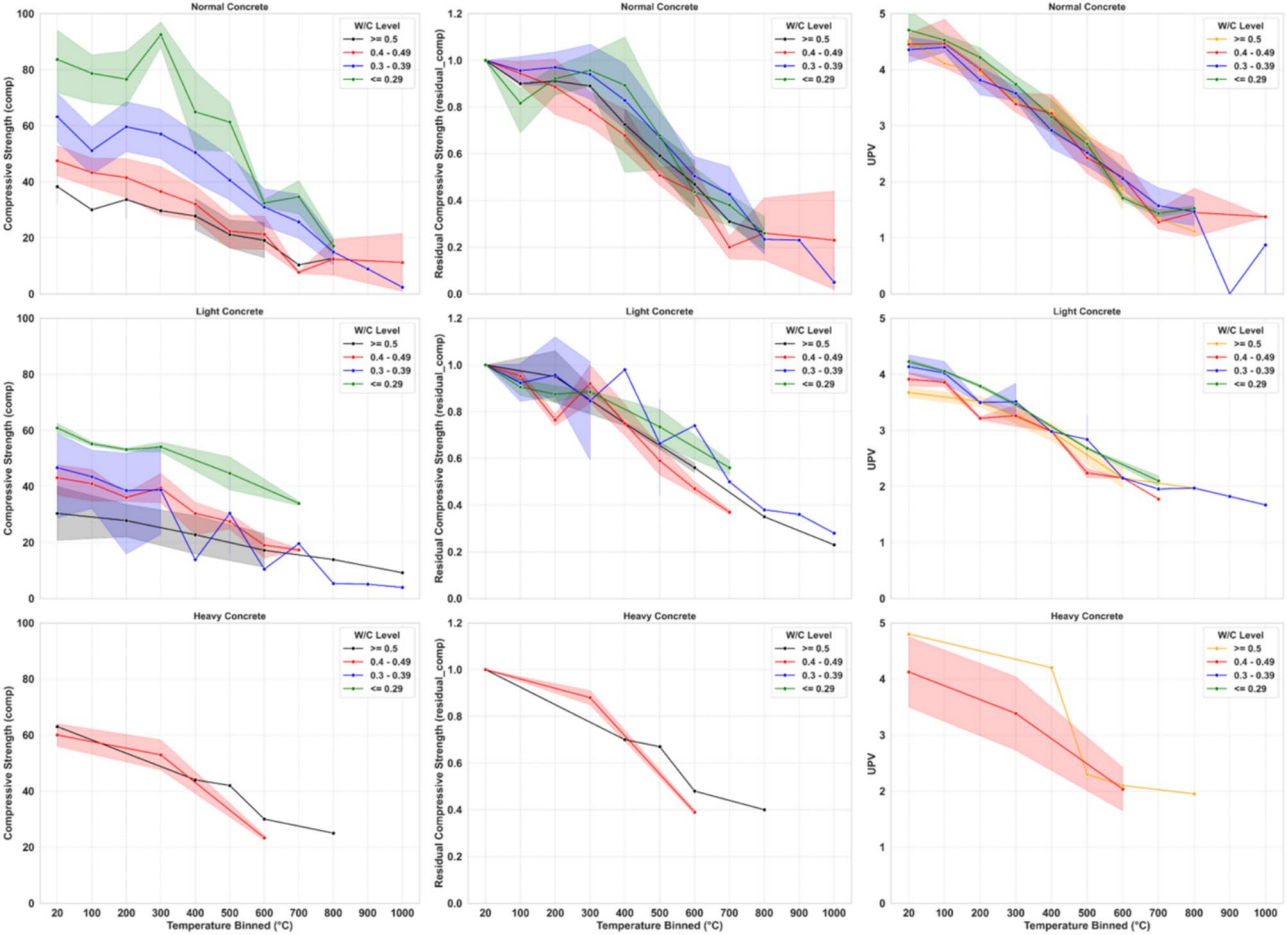
Analysis results of mechanical properties by temperature.

ANOVA analysis confirmed statistically significant differences (p < 0.05) in material properties based on concrete type (e.g., coarse aggregate density F=1788.60) and initial compressive strength based on W/C ratio (F=35.71), confirming the database comprises heterogeneous groups. The initial performance of concrete is highly non-linear. Figure 6 illustrates that degradation trends with temperature vary depending on concrete type and W/C ratio. This complexity limits simple statistical models and supports the necessity of an AI approach. Crucially, ANOVA results (Table 6) showed that while absolute compressive strength differed significantly (p < 0.05) across W/C groups (20–700℃), the residual compressive strength ratio did not show significant differences in the high temperature range. Crucially, ANOVA results showed that the residual strength ratio did not differ significantly in the high-temperature range (>500℃). Physically, beyond 500℃, the severe microcracking caused by calcium hydroxide decomposition and aggregate thermal expansion becomes the dominant degradation mechanism, overriding the influence of the initial pore structure determined by the W/C ratio.

**Table 6:** Results of descriptive statistics and ANOVA for each concrete type.

Temp (℃)	W/C : ≥ 0.5			W/C : 0.4 – 0.49			W/C : 0.3 – 0.39			W/C : ≤ 0.29			p-value		
	C	R.C	UPV	C	R.C	UPV	C	R.C	UPV	C	R.C	UPV	C	R.C	UPV
20	38.78	1.00	4.43	47.60	1.00	4.31	57.69	1.00	4.28	77.93	1.00	4.59	<0.05	-	0.28
100	30.01	0.90	4.11	42.13	0.95	4.16	46.65	0.94	4.18	69.30	0.85	4.34	<0.05	0.23	0.84
200	32.67	0.92	3.88	40.70	0.87	3.89	54.34	0.97	3.73	70.74	0.91	4.11	<0.05	0.38	0.33
300	29.67	0.89	3.44	39.89	0.85	3.34	47.88	0.89	3.55	77.12	0.93	3.63	<0.05	0.76	0.24
400	28.13	0.73	3.28	31.66	0.69	3.16	45.87	0.85	2.93	64.94	0.89	3.16	<0.05	0.08	0.47
500	24.15	0.60	2.65	23.84	0.53	2.37	35.47	0.67	2.68	55.74	0.70	2.68	<0.05	0.17	0.32
600	19.54	0.48	1.91	21.09	0.44	2.09	28.37	0.53	2.07	32.41	0.44	1.71	0.08	0.51	0.62
700	10.34	0.31	1.34	11.55	0.27	1.48	23.37	0.45	1.72	34.40	0.44	1.66	<0.05	0.08	0.53
800	14.38	0.29	1.33	12.35	0.26	1.45	13.35	0.26	1.55	17.03	0.26	1.53	0.90	0.97	0.83
900	-	-	-	-	-	-	6.99	0.30	0.91	-	-	-	-	-	-
1000	9.20	0.23	1.67	11.21	0.23	1.38	2.77	0.11	1.07	-	-	-	0.46	0.71	0.69

This suggests the rate of degradation may follow a more universal mechanism. Therefore, the residual compressive strength ratio, which normalizes initial conditions, is statistically more homogeneous and suitable as a dependent variable^11,12,18,57^. UPV also showed stability across W/C groups, indicating it reflects internal damage rather than initial mix conditions. The EDA strongly supports adopting the residual compressive strength ratio and UPV as key variables.

**Fig. 7:**
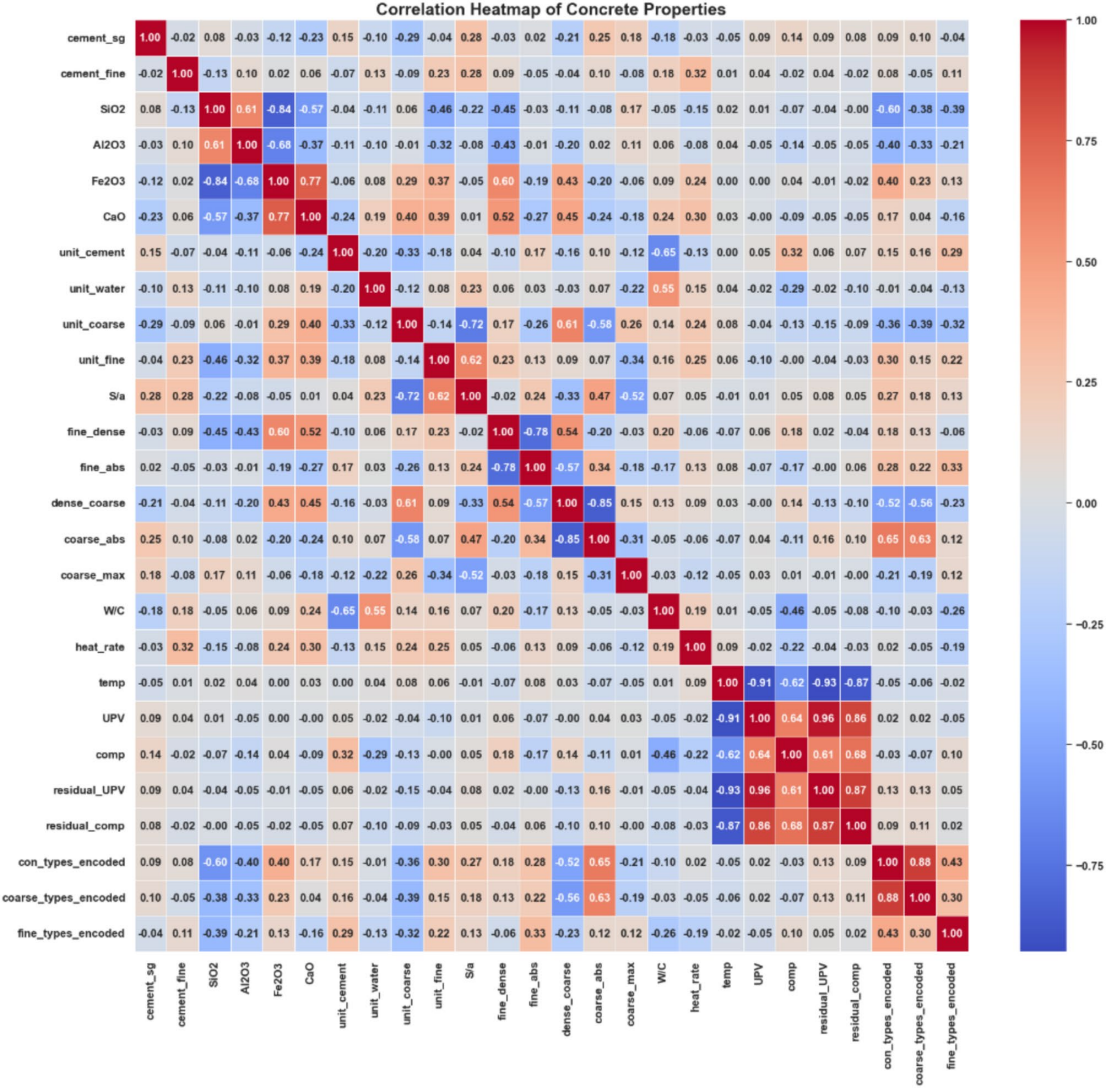
Correlation analysis results.

Correlation analysis (Fig. 7) identified key variables for modeling. The residual compressive strength ratio showed a strong negative correlation with temperature (r = -0.87) and a strong positive correlation with UPV (r = 0.86). This aligns with physical phenomena and confirms these three variables are the crucial indicators for quality evaluation^58^.

Furthermore, feature importance analysis confirmed that heterogeneity factors such as heating regimes and aggregate type had negligible impact (importance score < 0.05) on the residual strength ratio compared to the dominant variables of temperature and UPV. Note that specimen geometry was not included in the correlation analysis, as the utilization of the residual strength ratio, representing the relative proportion of thermal degradation, renders the absolute size effect negligible. This supports the validity of integrating data from diverse sources.

Conversely, the residual ratio showed almost no correlation with material/mix variables (e.g., W/C r = -0.08). While materials determine initial performance, their influence is diluted by temperature during degradation^3,20^. This validates focusing modeling on the dominant physical indicators: temperature, UPV, and residual strength^59^.

### Data augmentation and validation

**Fig. 8:**
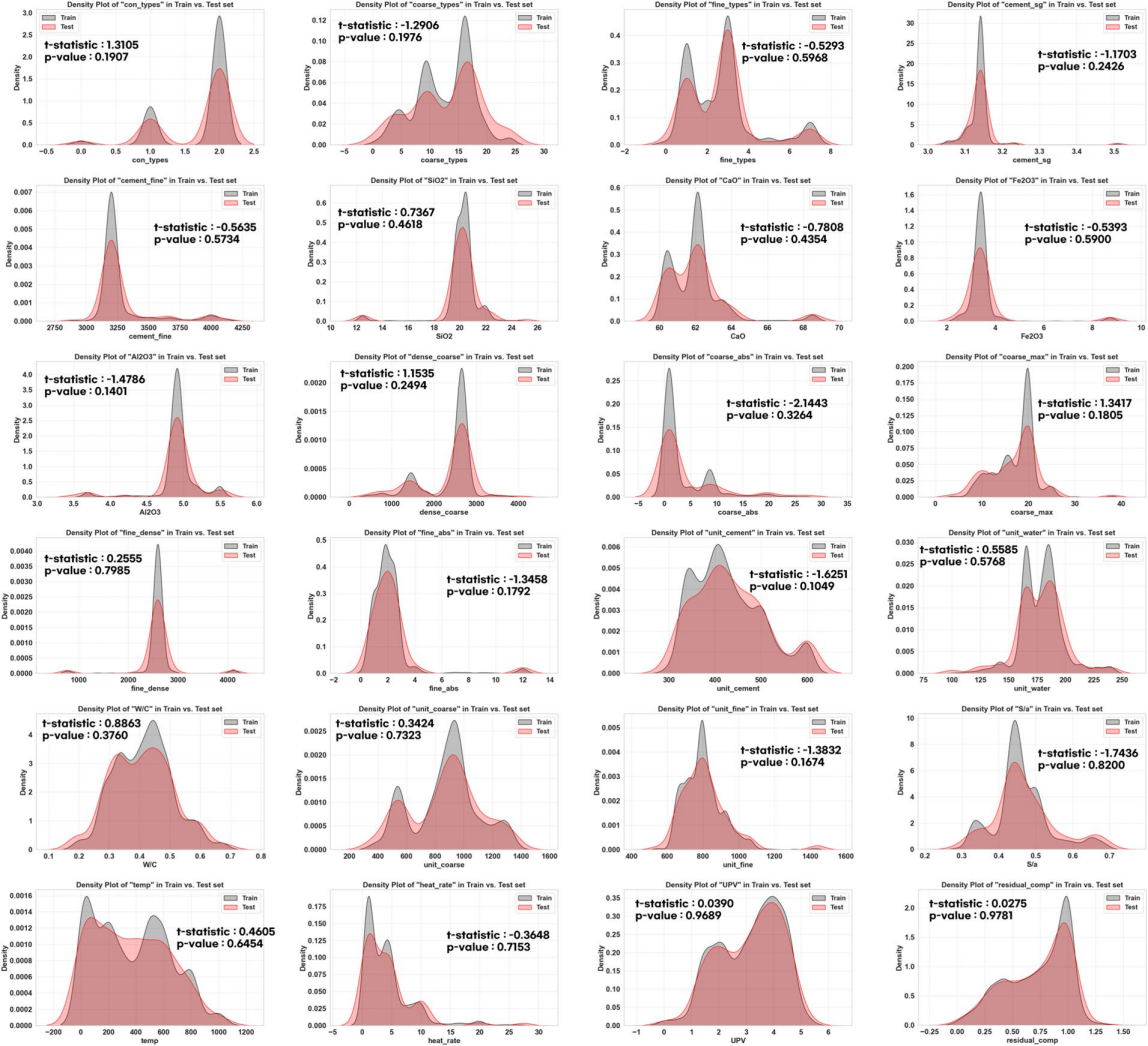
Distribution and significance test results of augmented data and original data.

To overcome data limitations and ensure robustness, data augmentation was performed using SMOTE. Statistical homogeneity between the original (334 points) and augmented (3,006 points) data was verified. Figure 8 compares the distributions and shows independent samples t-test results. The analysis showed p-values exceeded 0.05 for all variables, confirming the augmented data maintains the statistical characteristics of the original data without distortion. This augmentation provided abundant training data for the AI model to learn complex behavior, minimize overfitting, and ensure consistent predictive performance.

### Establishment of AI-driven quality standards

**Fig. 9:**
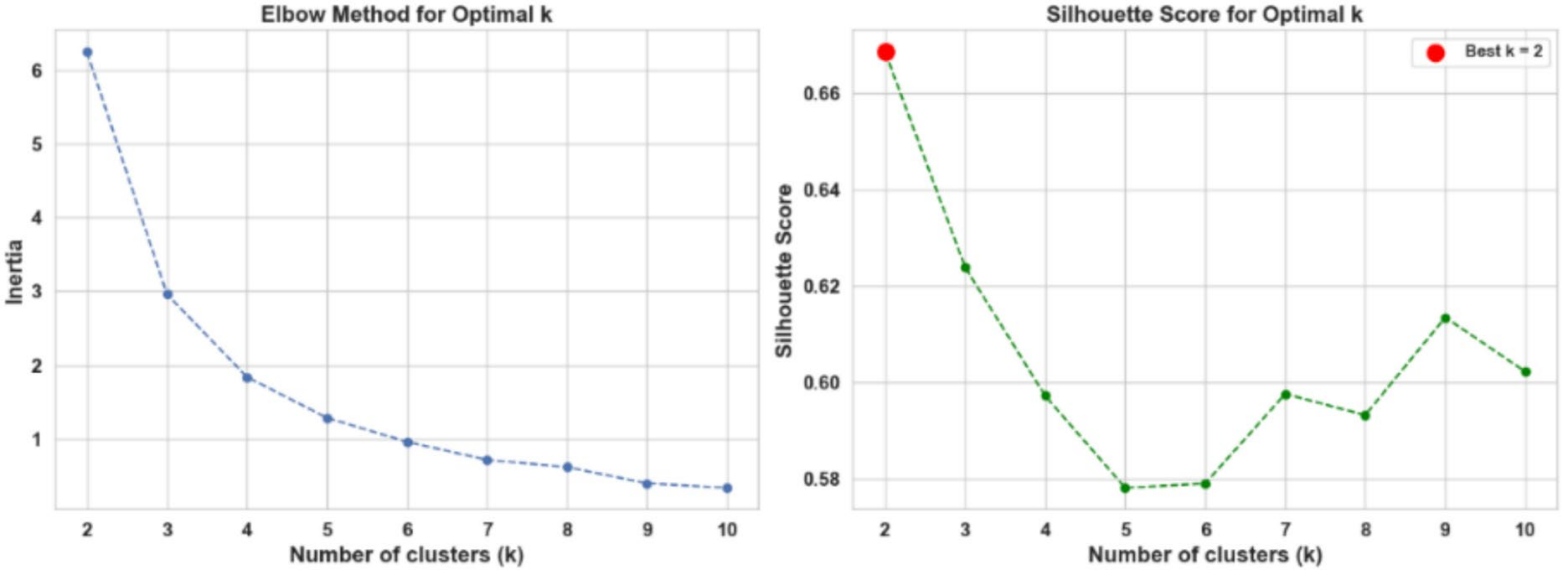
Elbow and Silhouette analysis results for setting the optimal number of clusters.

A hybrid AI approach was introduced to establish objective quality criteria, replacing reliance on inadequate room temperature standards. K-Means clustering was performed using the key variables: residual compressive strength ratio, temperature, and UPV. The optimal number of clusters (k) was determined using the elbow method (WCSS) and silhouette score (Fig. 9). The WCSS decrease rate slowed significantly beyond k=4 (elbow point, Fig. 9 left). The silhouette score (Fig. 9, right) was high at k=4 (approx. 0.60), providing practical segmentation corresponding to ‘Safety’, ‘Caution’, ‘Warning’, and ‘Danger’. Thus, k=4 was selected as optimal. The choice of k=4 was validated not only statistically but also practically, as it maps directly to actionable engineering levels (Safety, Caution, Warning, Danger). While K-Means acts as an initial labeling tool, the complex, non-linear boundaries of the degradation mechanism are effectively captured by the subsequent supervised learning models trained on these labels.

After labeling the data via clustering, a DT model was trained. The maximum tree depth (max_depth) was optimized to prevent overfitting.

**Fig. 10:**
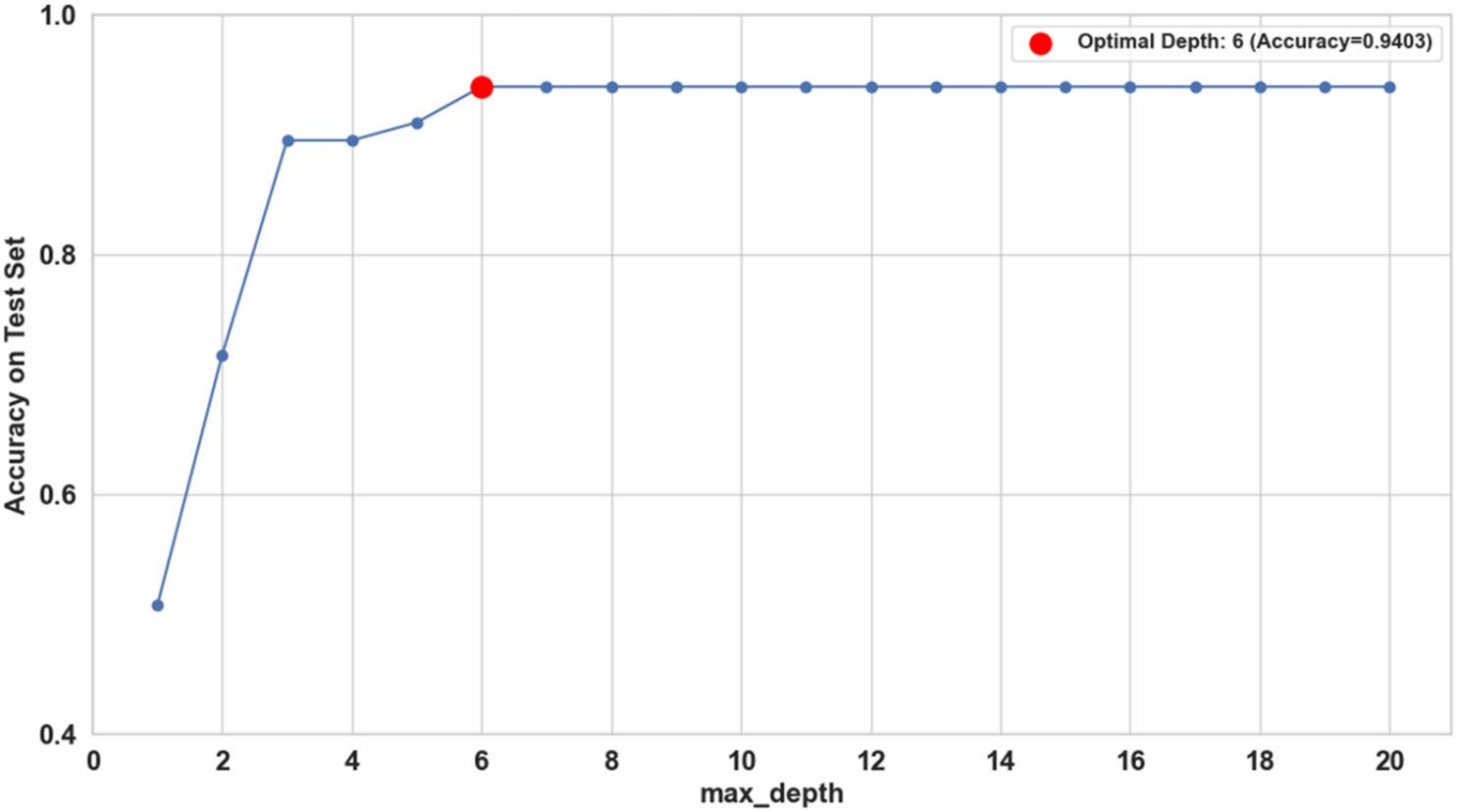
Evaluation results of optimal Max_depth.

**Fig. 11:**
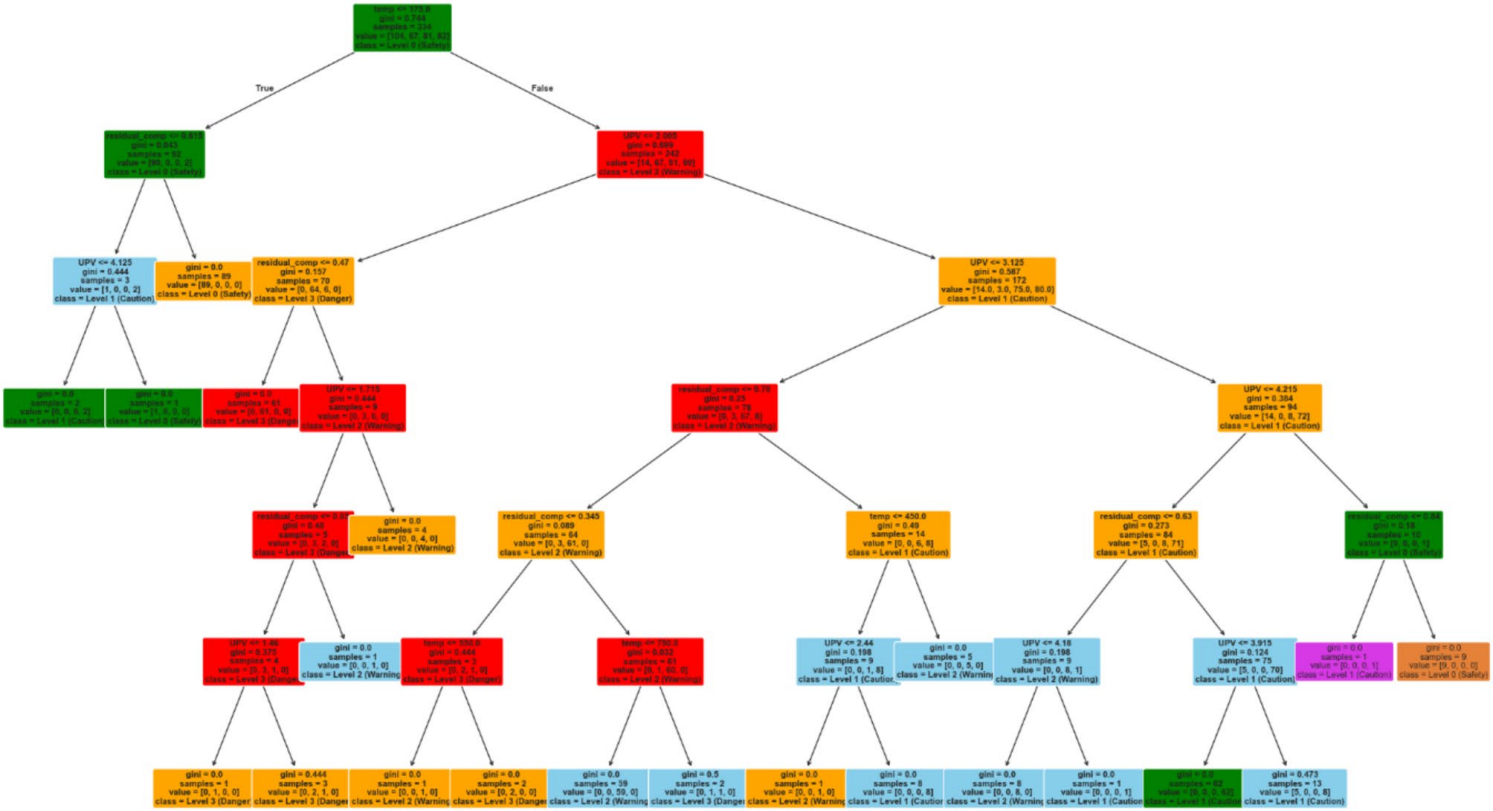
Setting concrete quality evaluation rules based on DT.

Figure 10 shows accuracy converging at max_depth=6 (accuracy 0.94), indicating the optimal balance between complexity and performance. The final rule-based model was constructed with max_depth=6. The optimized DT model rules are visualized in Fig. 11 and organized into 18 interpretable If-Then rules (Table 7). It is important to note that Table 7 visualizes the internal decision logic of the AI model to demonstrate transparency. It is not designed as a manual checklist for practitioners; instead, the AI model automatically applies these rules to classify data.

**Table 7:** Criteria for determining the quality of concrete after high-temperature exposure.

Rule No.	Condition 1	Condition 2	Condition 3	Condition 4	Final quality
1	temp <= 175	residual_comp <= 0.81	UPV <= 4.12	-	Caution
2	temp <= 175	residual_comp <= 0.81	UPV > 4.12	-	Safety
3	temp <= 175	residual_comp > 0.81	-	-	Safety
4	temp > 175	UPV <= 1.72	residual_comp <= 0.65	-	Danger
5	temp > 175	UPV <= 1.72	residual_comp > 0.65	-	Warning
6	temp > 175	1.72 < UPV <= 2.00	residual_comp > 0.47	-	Warning
7	temp > 175	2.00 < UPV <= 3.12	residual_comp <= 0.34	temp <= 550	Warning
8	temp > 175	2.00 < UPV <= 3.12	residual_comp <= 0.34	temp > 550	Danger
9	temp > 175	2.00 < UPV <= 3.12	0.34 < residual_comp <= 0.78	temp <= 750	Warning
10	temp > 175	2.00 < UPV <= 3.12	0.34 < residual_comp <= 0.78	temp > 750	Danger
11	temp > 175	2.00 < UPV <= 2.44	residual_comp > 0.78	temp <= 450	Warning
12	temp > 175	2.44 < UPV <= 3.12	residual_comp > 0.78	temp <= 450	Caution
13	temp > 450	2.00 < UPV <= 3.12	residual_comp > 0.78	-	Warning
14	temp > 175	3.12 < UPV <= 4.18	residual_comp <= 0.63	-	Warning
15	temp > 175	4.18 < UPV <= 4.21	residual_comp <= 0.63	-	Caution
16	temp > 175	3.12 < UPV <= 4.21	residual_comp > 0.63	-	Caution
17	temp > 175	UPV > 4.21	residual_comp <= 0.84	-	Caution
18	temp > 175	UPV > 4.21	residual_comp > 0.84	-	Safety

**Fig. 12:**
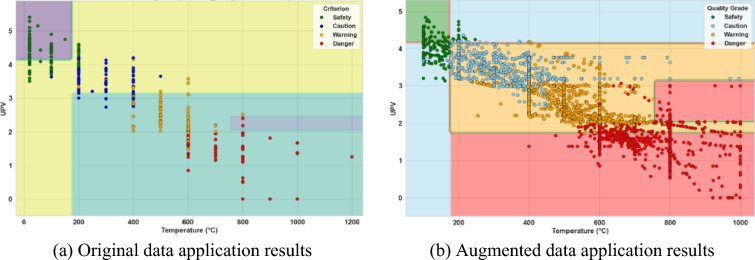
Visualization of the established quality classification criteria.

For example, Rule No. 4: ‘If the temperature exceeds 175°C, and UPV is 1.72 km/s or less, and the residual compressive strength ratio is 0.65 or less, then the final quality is Danger grade’. This provides a quantitative standard replacing existing unclear criteria. Figure 12 shows the application of these criteria to the original (a) and augmented (b) data. The grades are clearly distinguished according to physical phenomena: Safety in low-temp/high UPV areas, and Danger in high damage areas.

The augmented data (b) visualizes the decision boundary, demonstrating the robustness of the criteria. The dataset augmentation process adjusted the class distribution as follows: the raw dataset contained 99 Safety, 87 Caution, 78 Warning, and 70 Danger samples, while the augmented dataset now consists of 163 Safety, 750 Caution, 881 Warning, and 878 Danger samples.

### Model performance evaluation

**Fig. 13:**
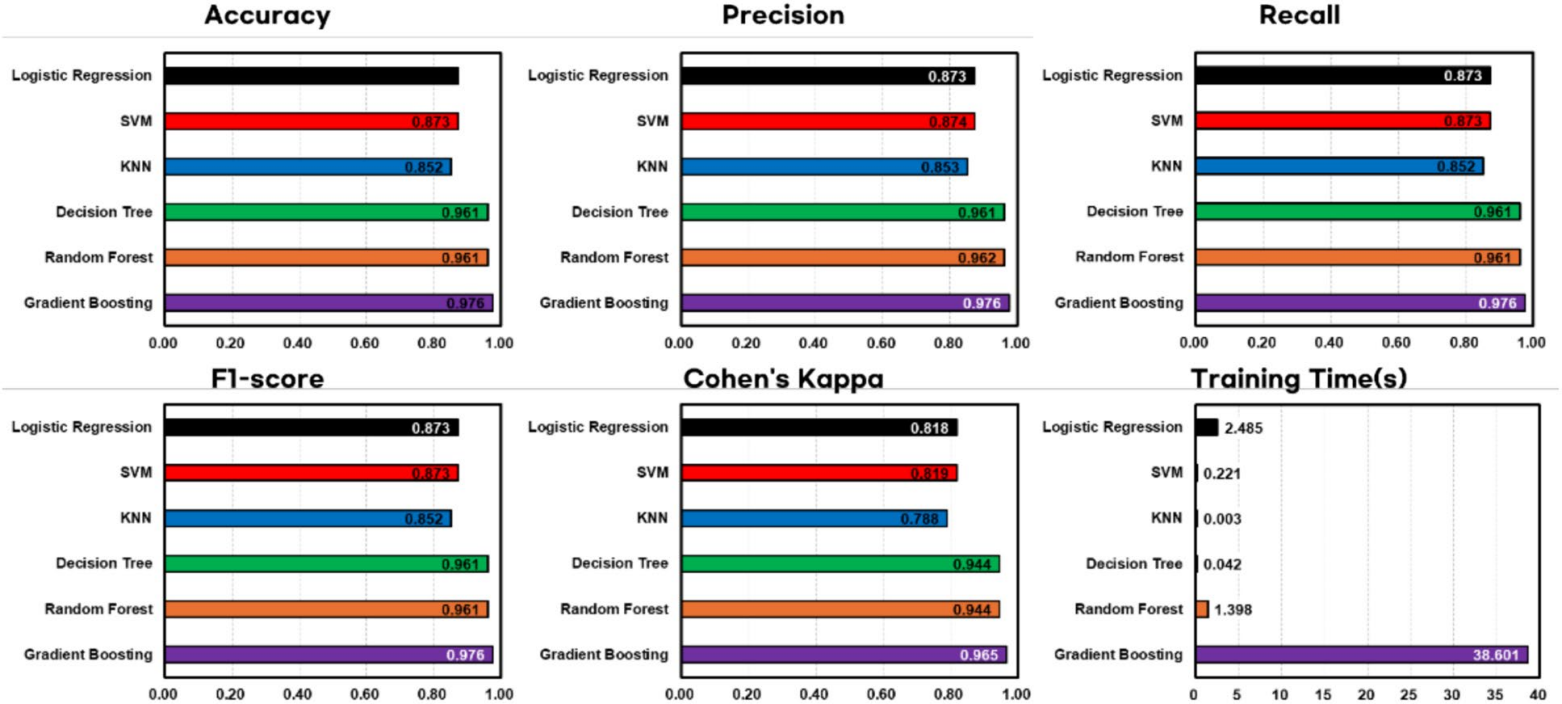
Model performance evaluation results.

Six supervised learning classification models (LR, SVC, KNN, DT, RF, GB) were constructed using the established quality criteria (4 grades). Their performance was evaluated using standard metrics^60^. Figure 13 summarizes key performance indicators. Ensemble techniques (RF, GB) and DT showed superior performance. GB achieved the best overall performance (accuracy 0.976, f1-Score 0.976). DT and RF also recorded high accuracy (0.961). LR, SVM, and KNN showed lower performance (accuracy 0.85–0.87). In cohen’s kappa, GB showed near perfect agreement (0.968).

**Fig. 14:**
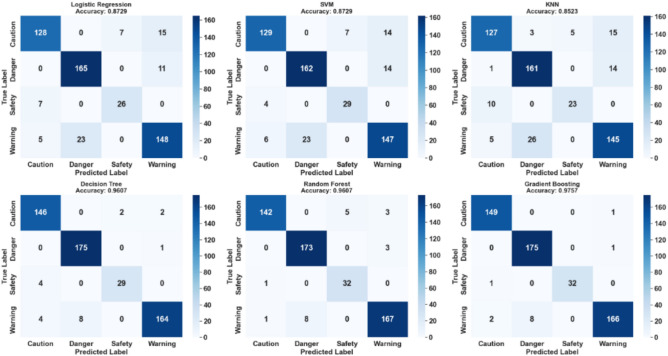
Confusion matrix evaluation results.

Training time analysis revealed a trade-off. KNN (0.003s) and DT (0.042s) were fastest, while GB (highest accuracy) was slowest (38.601s). The confusion matrix (Fig. 14) details classification accuracy per class. GB misclassified only 13 out of 534 test points and perfectly classified the critical Danger grade. DT also showed stable performance (21 misclassifications). KNN had 79 misclassifications (85.2% accuracy), showing confusion between adjacent grades^61^.

**Fig. 15:**
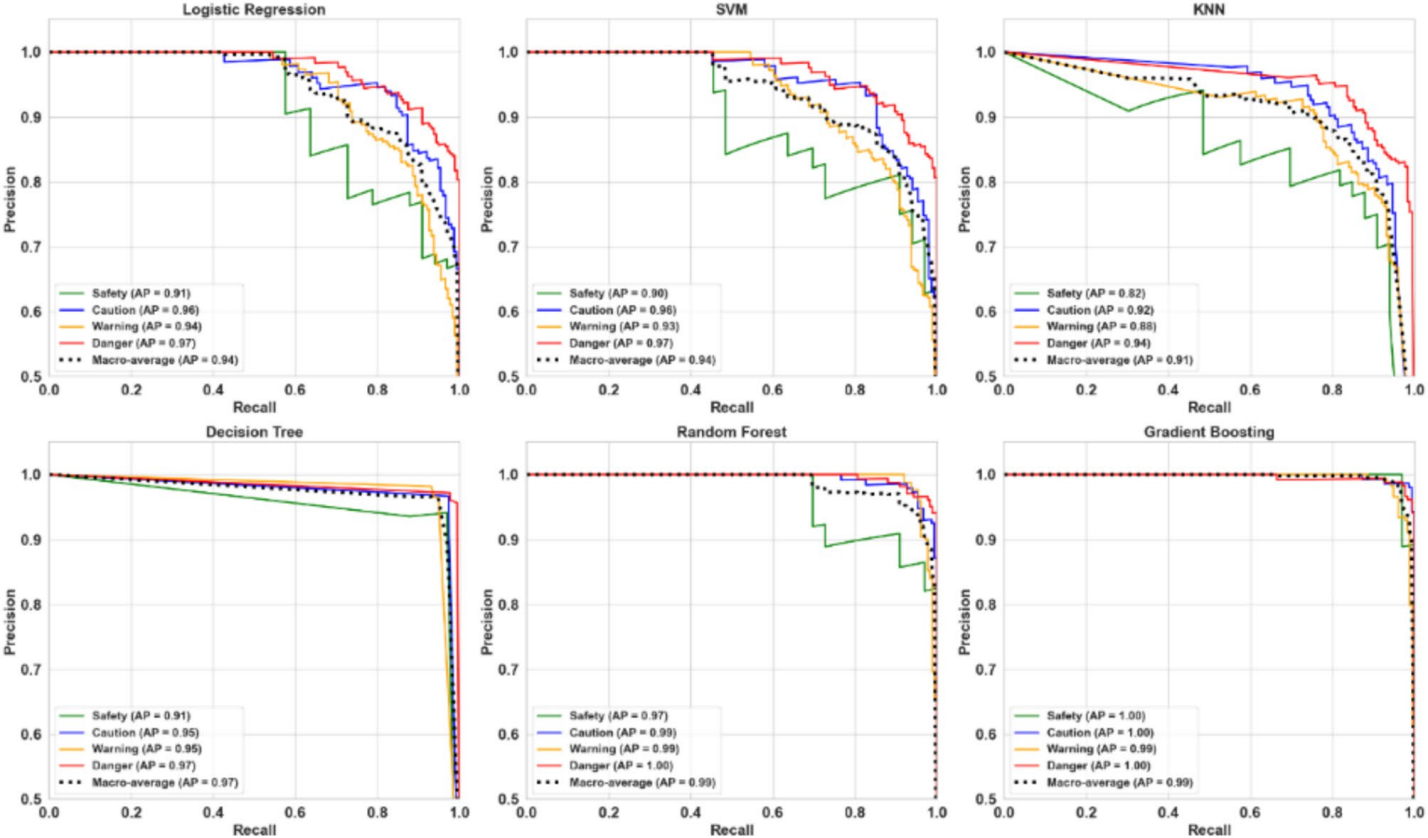
Precision-recall curve analysis results.

**Fig. 16:**
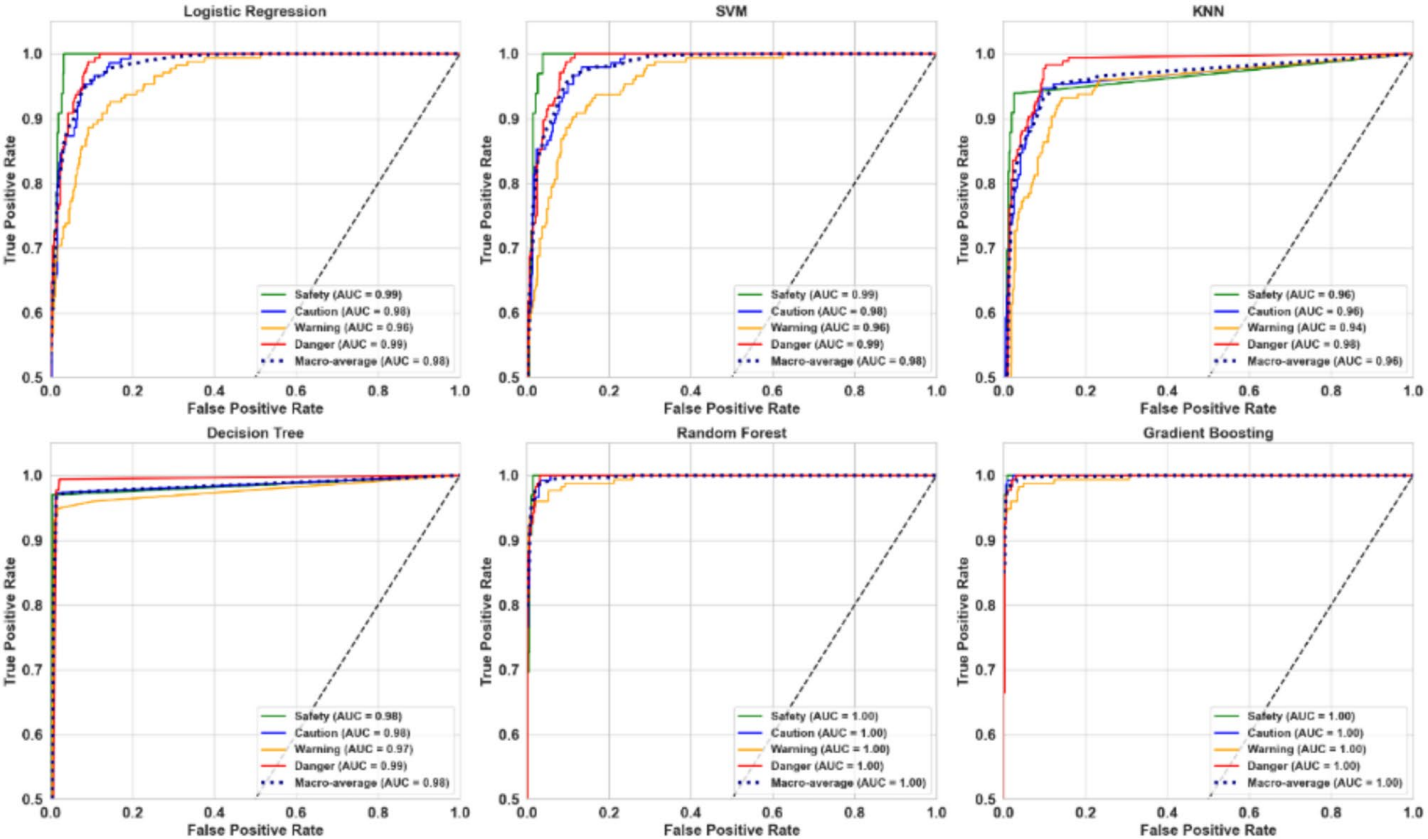
ROC and AUC evaluation results.

Generalization performance and reliability were further evaluated. The recision-recall curve (Fig. 15) evaluates performance on imbalanced classes. GB and RF showed near-perfect performance (AP ≈ 1.00) in all classes (macro-average AP 0.99 and 0.98). DT also showed high performance (0.97). The ROC curve (Fig. 16) indicates class distinction ability. RF showed perfect classification (AUC=1.00). GB and DT also showed excellent performance (0.98).

### Model interpretation and physical relevance

**Fig. 17:**
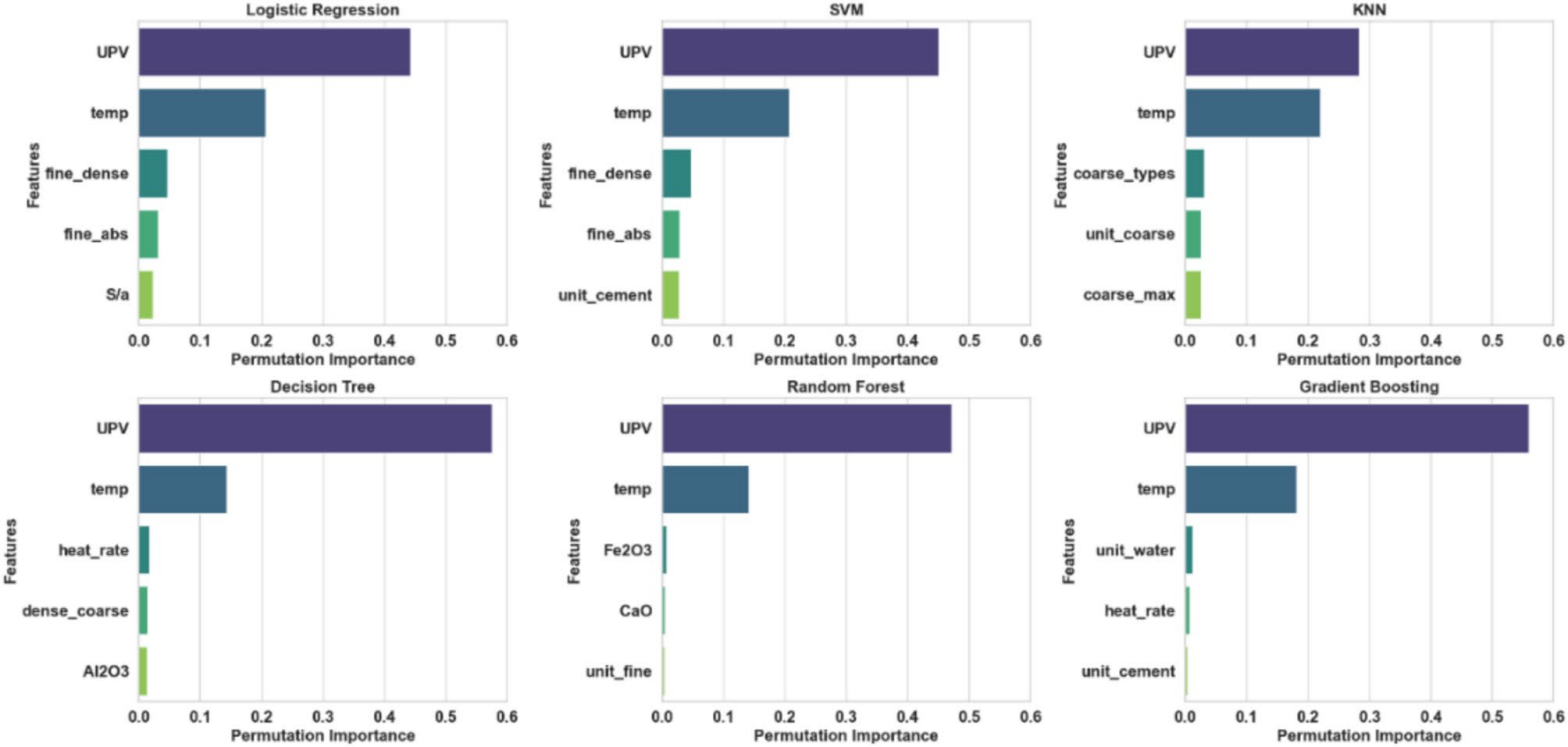
Variable importance evaluation results.

To understand the basis of the models’ predictions, variable importance, decision boundaries, and partial dependence were analyzed. Variable importance analysis (Fig. 17) confirmed UPV and temperature as the dominant variables across all models. In the GB model, UPV (0.56) and temperature (0.18) accounted for approximately 74% of the prediction. This aligns with the EDA findings, suggesting the models successfully learned the physical mechanisms of concrete degradation^62,63^.

**Fig. 18:**
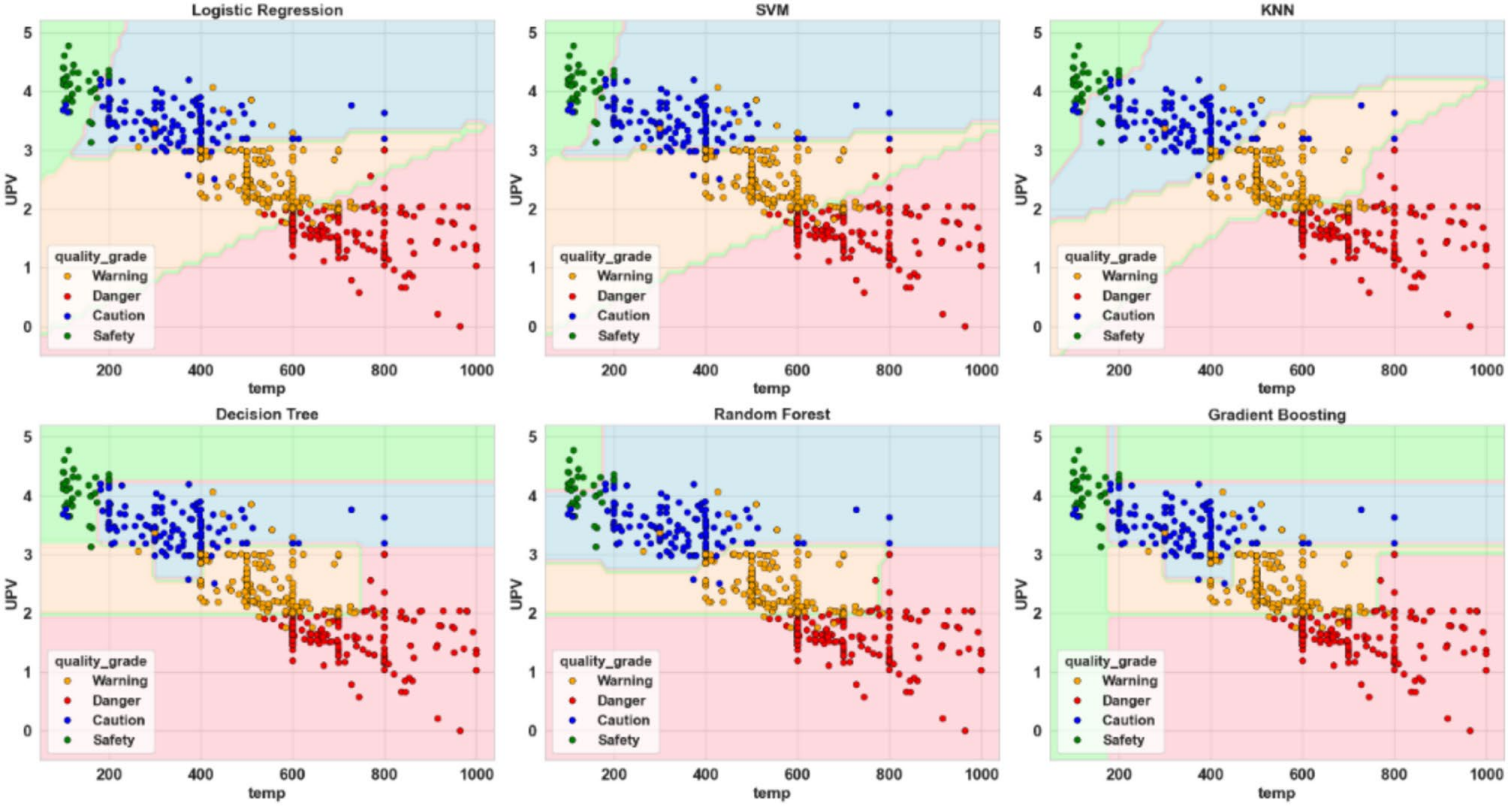
Decision boundary evaluation results.

Visualization of the decision boundaries (Fig. 18) illustrates how different algorithms separate the feature space. DT, RF, and GB formed complex, non-linear boundaries, precisely distinguishing the quality grades. In contrast, the simpler boundaries of LR or SVM struggled to capture these complex interactions. This indicates that tree based models are better suited for modeling the complex degradation mechanisms of concrete exposed to high temperatures.

**Fig. 19:**
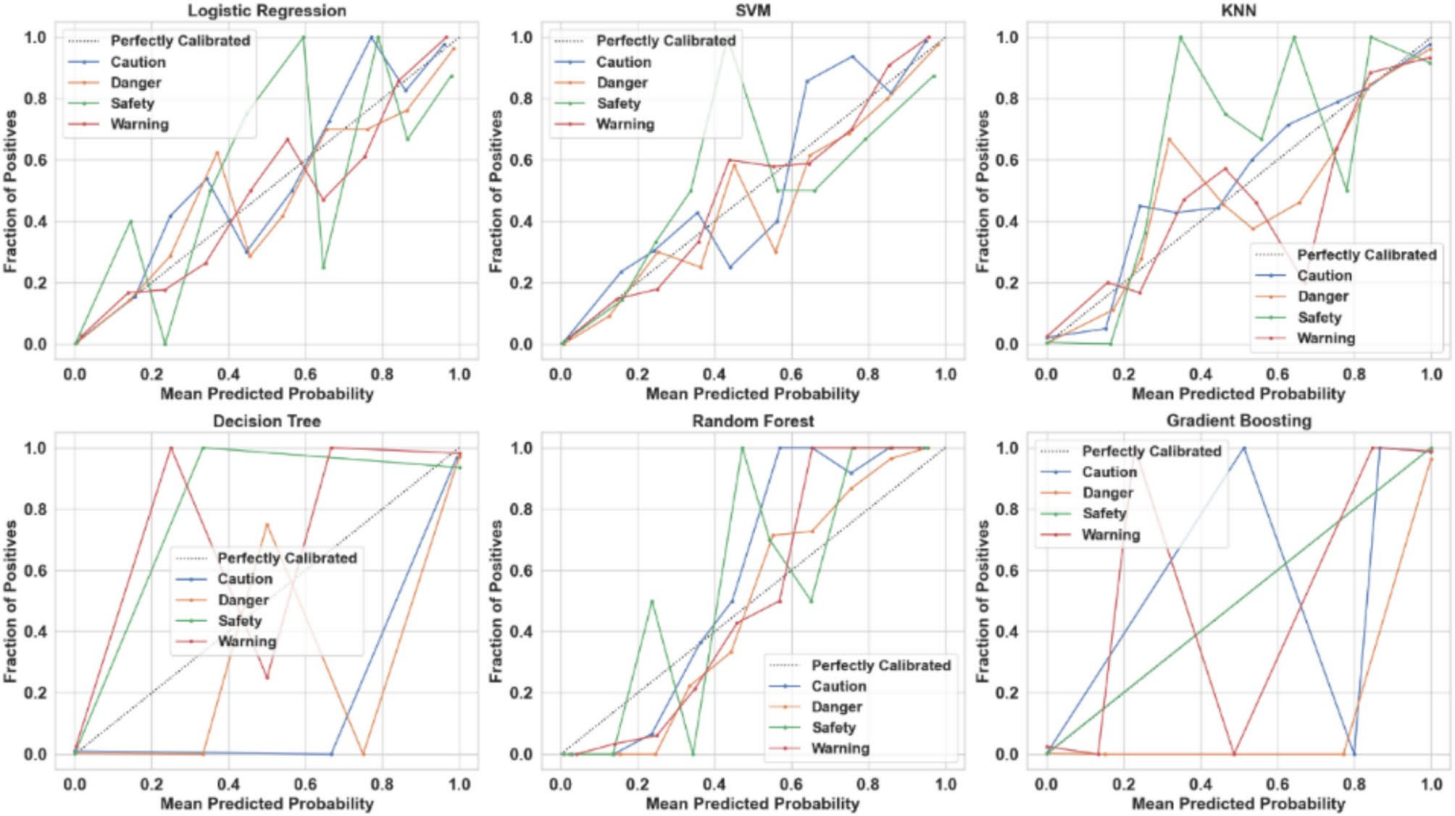
Calibration curve analysis results.

**Fig. 20:**
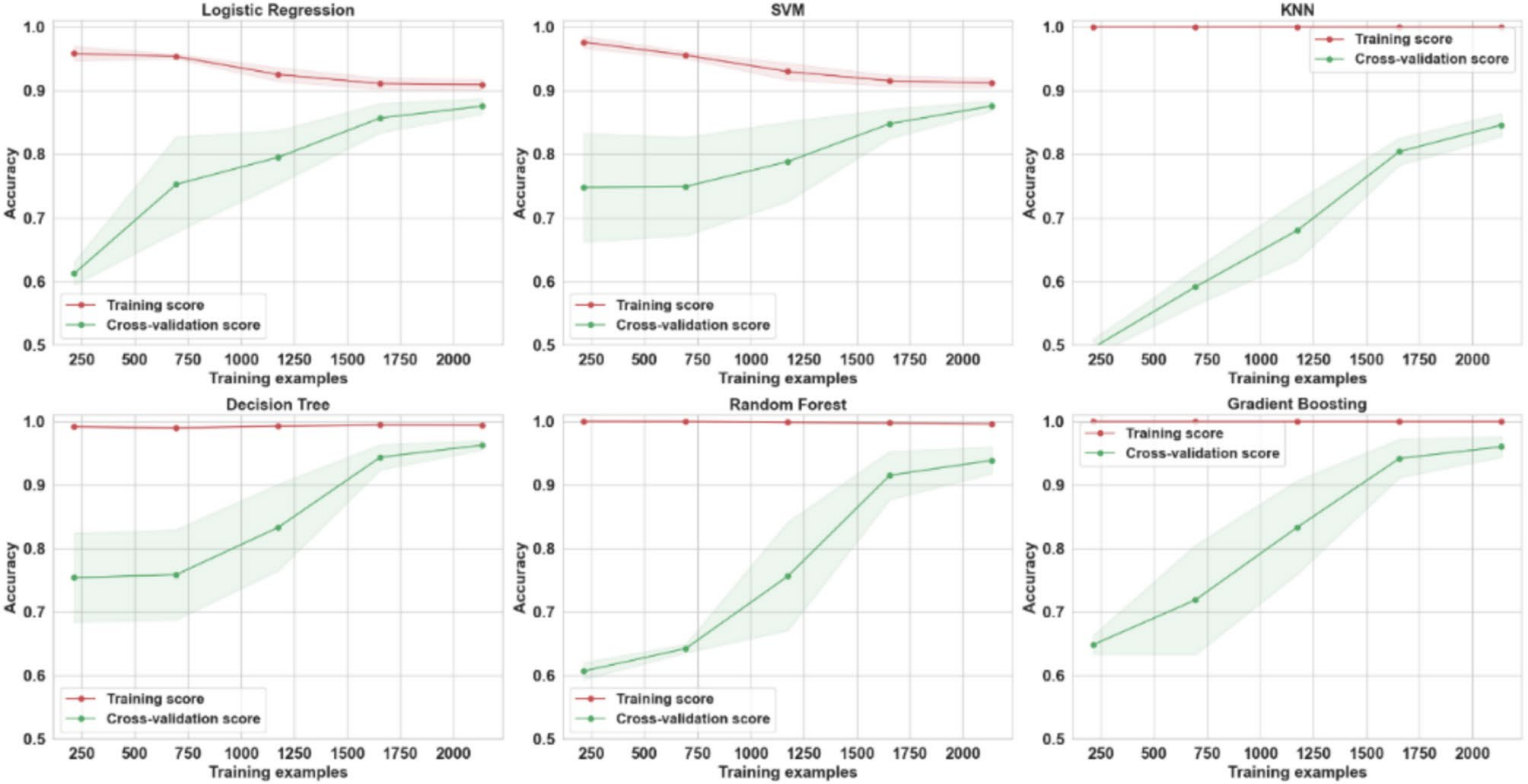
Learning curve analysis results.

Model reliability was assessed using calibration curves (Fig. 19) and learning curves (Fig. 20). DT and LR showed excellent calibration, while RF and GB showed slight overconfidence. For GB, RF, and DT, training and cross validation scores converge at a high level with narrow gaps, indicating an ideal learning state without overfitting^64^.

**Fig. 21:**
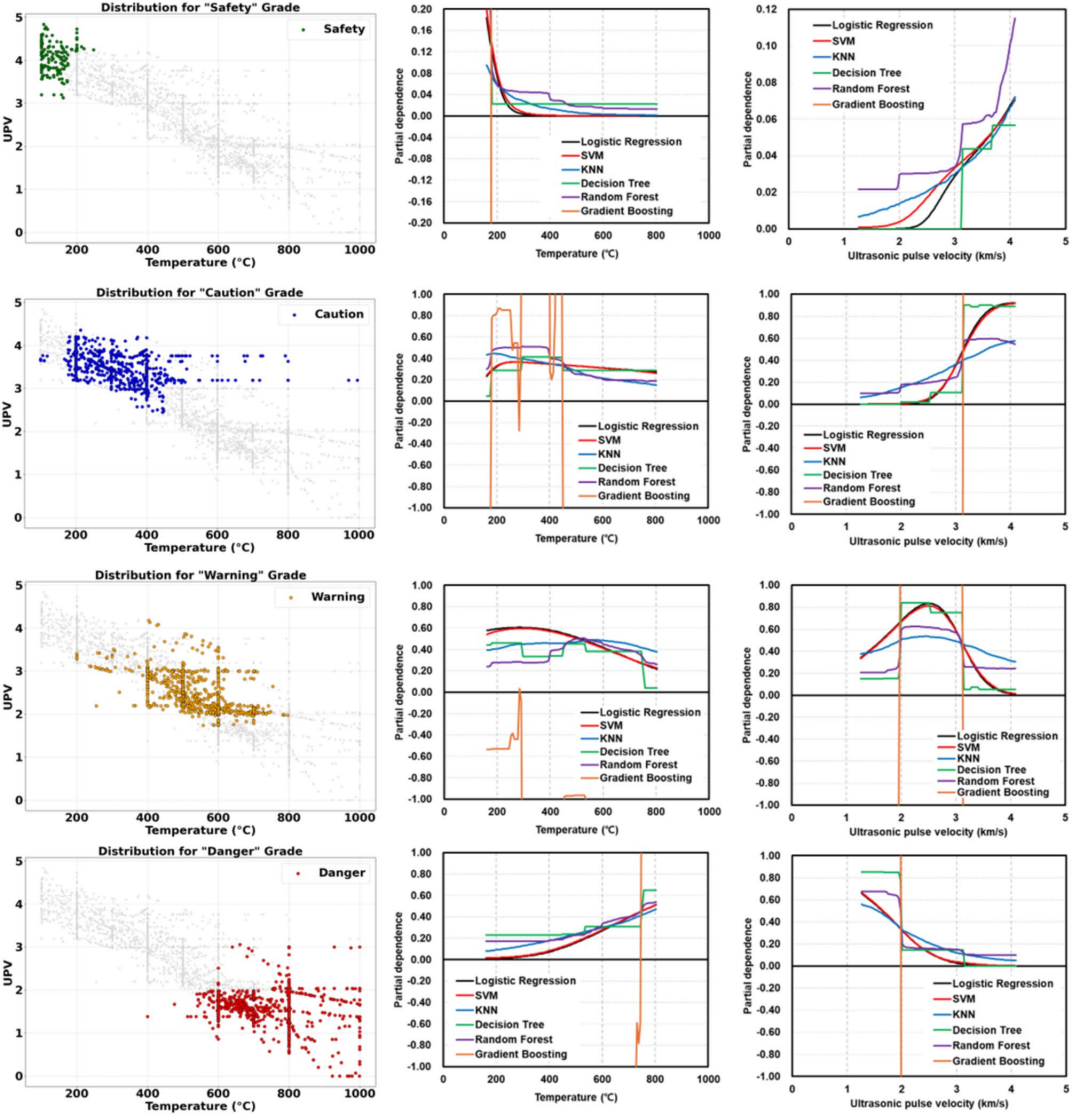
PDP analysis results by quality.

The partial dependence plot (PDP) (Fig. 21) further illustrates the marginal impact of the key variables on the classification probability. In all models, the probability of the Danger grade increased significantly as temperature increased and UPV decreased. Conversely, the Safety grade showed the opposite trend. This confirms that the models’ learning is physically valid and reflects the expected behavior of fire-damaged concrete.

### Optimal model selection and practical implications

The comprehensive evaluation synthesized accuracy, stability, reliability, interpretability, and efficiency (Table 8). While GB model achieved the highest accuracy (97.6%), the DT model obtained the highest overall score (4.0). DT maintained high accuracy (96.1%) while offering decisive advantages in computational efficiency and interpretability (5 points each).

**Table 8:** Comparative evaluation of model performance.

Model	Performance	Stability	Reliability	Interpretability	Efficiency	Overall
Logistic regression	Average(3)	Good(4)	Excellent(5)	Good(4)	Average(3)	3.8
SVM	Average(3)	Good(4)	Good(4)	Good(4)	Good(4)	3.8
KNN	Fair(2)	Average(3)	Fair(2)	Fair(2)	Excellent(5)	2.8
Decisiontree	Good(4)	Average(3)	Average(3)	Excellent(5)	Excellent(5)	4.0
Randomforest	Good(4)	Good(4)	Average(3)	Average(3)	Good(4)	3.6
Gradient boosting	Excellent(5)	Excellent(5)	Average(3)	Average(3)	Poor(1)	3.4

In the context of structural safety diagnosis of fire-damaged structures, it is crucial for engineers to understand the basis for the model’s judgment. Black-box models, such as GB or RF, offer high accuracy but are difficult to interpret internally. Conversely, DT is a white-box model that provides the classification process as clear, explicit rules (as demonstrated in Table 7). This transparency is essential for securing trust and supporting responsible engineering decision-making in the field. Therefore, the DT model is proposed as the optimal AI framework for this application.

It strikes the necessary balance between excellent predictive performance and the efficiency and interpretability required for rapid, reliable field assessments. The hybrid AI methodology maximizes reliability by replacing outdated criteria with data-driven standards, providing high utility for structural safety diagnosis and repair decisions. Although the proposed classification is data-driven, the derived Danger threshold aligns with the severe strength loss regions defined in ACI 216 and Eurocode 2, demonstrating high consistency with established engineering consensus despite the absence of specific UPV-based standards.

## Conclusion

This study developed a novel AI-driven quality classification framework to address the limitations of traditional methods in evaluating the residual performance of concrete exposed to high temperatures. By integrating advanced computational techniques, specifically hybrid ML and data augmentation, we addressed critical challenges in the field of structural integrity assessment, namely data scarcity and the lack of objective, specialized quality criteria.

The primary contributions and findings of this research are:

1. We successfully implemented a framework that shifts the research paradigm from numerical prediction to actionable quality classification, outputting grades such as ‘Safety’, ‘Caution’, ‘Warning’, and ‘Danger’ that are directly linked to engineering decision making. This approach moves beyond traditional regression-centric methods, effectively bridging the prediction-decision gap and significantly enhancing practical applicability for infrastructure evaluation.
2. A hybrid AI approach combining unsupervised K-Means clustering and supervised DT learning was utilized to derive 18 objective, interpretable, and rule based classification criteria. This methodology provides a robust foundation for establishing data-driven standards specialized for fire damaged concrete, effectively replacing subjective or inappropriate criteria based on room-temperature conditions.
3. To address the inherent data scarcity in high-temperature experiments, the SMOTE algorithm was employed to expand the dataset from 334 to 3,006 points. This computational solution significantly enhanced model robustness, minimized the risk of overfitting, and ensured high generalization performance in modeling the complex, non-linear degradation of concrete.
4. A systematic comparison of six machine learning models identified the Decision Tree as the optimal model for field application, achieving an accuracy of 96.1%. Although GB demonstrated slightly higher accuracy (97.6%), the DT was selected for its superior computational efficiency and crucial white-box interpretability, which ensures trust and reliability in safety critical applications where UPV and exposure temperature act as the dominant factors.
5. For practical implementation, a site engineer would utilize this framework through the following concrete scenario. First, to ensure measurement accuracy, strict calibration of the UPV device using a standard reference block must be performed prior to inspection. Next, the engineer measures UPV across the structural grid while simultaneously estimating exposure temperatures (e.g., via concrete colorimetry). Crucially, instead of relying on destructive core sampling, the residual strength ratio is first predicted based on these measured UPV and temperature values, utilizing the correlation models established in this study. Finally, by inputting these three parameters, measured UPV, estimated temperature, and the predicted residual strength ratio, into the proposed 18 ‘If-Then’ rules (Table 8), the engineer can immediately derive an objective quality grade (‘Safety’ to ‘Danger’). This process enables rapid, non-destructive decision-making for structural repairs.

By providing a reliable, objective method to assess fire-damaged concrete, this framework significantly advances the tools available for structural assessment and supports sustainable infrastructure management by enabling informed decisions on structural repair and reuse.

## Limitations and future work

While the proposed AI-driven framework demonstrates high utility, several limitations must be acknowledged. First, the database relies on heterogeneous literature sources. Variations in experimental conditions (e.g., heating/cooling rates, specimen sizes) are not fully parameterized, introducing potential uncertainty. Methodologically, while SMOTE addressed data scarcity, synthetic data may not entirely capture complex physical behaviors. Furthermore, the quality grades derived from K-Means clustering are inherently dependent on the dataset’s structure rather than established engineering consensus.

Second, a significant gap exists between the controlled laboratory data used for training and real-world fire scenarios. The training data utilized uniformly heated, unloaded specimens. Consequently, the model does not account for the effects of heterogeneous heating gradients, complex stress states under structural loads, spalling risks, or varying moisture content inherent in actual fires. Therefore, comprehensive field validation on real fire-damaged structures is essential to confirm the model’s reliability in practice.

Future research will focus on expanding the database to include various mineral admixtures and, crucially, to account for mechanical loading conditions during exposure, as the current dataset is primarily based on unloaded specimens. We also aim to integrate multi-modal NDT data (e.g., acoustic emission, thermal imaging). Extensive field validation on actual fire-damaged structures remains essential to confirm the model’s reliability in complex, real-world scenarios. Furthermore, to maximize practical utility, we plan to implement the proposed framework as a mobile based application. The user interface (UI) will be designed to allow intuitive input of field measurements (UPV and estimated temperature) and immediate visualization of quality grades. To support rapid decision-making, the platform will integrate tools such as QR code scanning for efficient data entry and location tracking, as well as automated report generation, ensuring seamless usability for site engineers.

## Data Availability

The raw data and Python scripts used to establish the properties of concrete after high-temperature exposure and develop the predictive model are publicly available in the Zenodo repository (10.5281/zenodo.19907194).
